# An investigation of the disparity in estimates of microfilaraemia and antigenaemia in lymphatic filariasis surveys

**DOI:** 10.1093/trstmh/trv048

**Published:** 2015-06-21

**Authors:** Jorge Cano, Paula Moraga, Birgit Nikolay, Maria P. Rebollo, Patricia N. Okorie, Emmanuel Davies, Sammy M. Njenga, Moses J. Bockarie, Simon J. Brooker

**Affiliations:** a Faculty of Infectious and Tropical Diseases, London School of Hygiene & Tropical Medicine, Keppel Street, London, UK; b NTD Support Center, Task Force for Global Health, Decatur, Atlanta, USA; c Institute for Advanced Medical Research and Training, College of Medicine, University of Ibadan, Ibadan, Nigeria; d Ministry of Health, Nigeria; e Eastern and Southern Africa Centre of International Parasite Control, Kenya Medical Research Institute (KEMRI), Nairobi, Kenya; f Centre for Neglected Tropical Diseases, Liverpool School of Tropical Medicine, Liverpool, UK

**Keywords:** Antigenaemia, Logistic regression, Lymphatic filariasis, Microfilaraemia, Prevalence surveys

## Abstract

**Background:**

The diagnosis of lymphatic filariasis (LF) is based typically on either microfilaraemia as assessed by microscopy or filarial antigenaemia using an immuno-chromatographic test. While it is known that estimates of antigenaemia are generally higher than estimates of microfilaraemia, the extent of the difference is not known.

**Methods:**

This paper presents the results of an extensive literature search for surveys that estimated both microfilaraemia and antigenaemia in order to better understand the disparity between the two measures.

**Results and Conclusions:**

In some settings there was a very large disparity, up to 40–70%, between estimates of microfilaraemia and antigenaemia. Regression analysis was unable to identify any predictable relationship between the two measures. The implications of findings for risk mapping and surveillance of LF are discussed.

## Introduction

The control and elimination of lymphatic filariasis (LF), a disfiguring and disabling mosquito-borne disease,^1^ necessitates a detailed understanding of the geographical distribution of infection. Mapping of LF has been completed in nearly all endemic countries and many countries are now undertaking transmission assessment surveys (TAS) in order to determine whether mass drug administration (MDA) can be stopped. Reliable diagnosis is an essential prerequisite for mapping, TAS and post-MDA surveillance. In the past, diagnosis was principally based on the use of blood films collected during night blood surveys to detect the presence of microfilariae (mf) by expert microscopy.^2^ The development, in the 1990s, of a simple and rapid antigen detection test for *Wuchereria bancrofti* antigenaemia, based on the immuno-chromatographic test (ICT), revolutionized LF surveys since it avoided the need for night blood surveys and time-consuming microscopy.^3^ It is well-known that estimates of antigenaemia are typically higher than estimates of microfilariae for a number of biological and practical reasons, including the fact that antifilarial drugs are highly effective against microfilariae but have minimal effect on adult worms.^4^ What is less clear is the extent of the disparity in estimates and whether prevalence of antigenaemia can be used to predict the mf prevalence, and vice versa. To investigate these issues, we analyzed the relationship between prevalence of LF based on antigenaemia using ICT and microfilaraemia using microscopy. The implications for risk mapping and surveillance of LF are discussed.

## Materials and methods

An exhaustive literature search was made to identify prevalence surveys where microfilaraemia and antigenaemia were assessed among the study population. The search included surveys according to previously defined inclusion and exclusion criteria.^5^ Studies were stratified based on whether they were conducted before the initiation of large-scale MDA (pre-intervention) or once MDA had been initiated, as part of monitoring activities (post-intervention). Studies were additionally stratified by continent in order to capture differences in mosquito species distribution and socio-economic conditions, type of ICT test (i.e., AMRAD vs Binax), and type of blood collected (venous blood vs peripheral blood). We assumed that Binax ICT was used in surveys conducted from 2001 when the type of ICT card test was not provided (n=74 surveys). In all identified surveys, mf diagnosis was based on night blood sampling. The relationship between the prevalence of microfilaraemia (p_mf_) and antigenaemia (p_ICT_) was fitted with logistic regression as follows:

$$\hbox{for each survey}\,i = 1, \ldots ,N,$$

$${Y_{m{f_i}}}|{p_{m{f_i}}} \sim Binomial\; ({{N_{m{f_i}}},{p_{m{f_i}}}} )$$

$$\eqalign{ \hbox{logit} ({p_{Mf_{i}}}{\rm )} = & \; \alpha + {\alpha _1}\hbox{logit}\, ( {p_{IC{T_i}}}) + {\alpha _2}\hbox{logit}\,{ ( {p_{IC{T_i}}} ) ^2} + {\alpha _{{PRE}}} \times PR{E_i} \cr & + {\alpha _{1PRE}}\hbox{logit}\, ({p_{IC{T_i}}}) \times PR{E_i} + {\alpha _{2PRE}}\hbox{logit}\,({p_{IC{T_i}}}) ^2 \times PR{E_i} \cr & + {\alpha _{AMERICAS}} \times AMERICA{S_i} + {\alpha _{ASIA}} \times ASI{A_i} \cr & + {\alpha _{OCEANIA}} \times OCEANI{A_i} + {\alpha _{TYPECIT\_}} \times TYPECI{T_i} \cr & + {\alpha _{VENOUSBLOOD\_}} \times VENOUSBLOO{D_i},} $$

where ${Y_{m{\,f_i}}}$ and ${N_{m{\,f_i}}}$ denote the number of positive individuals and the total number of individuals tested by parasitological techniques (either the thick smear or filtration method), respectively. ${\,p_{IC{T_i}}}$is the prevalence estimated using the ICT and $PR{E_i}\; $is a binary variable that is equal to 1 if the observation corresponds to a survey conducted in pre-control settings and 0 to post-control settings. $AMERICA{S_i}$, $ASI{A_i}$ and $OCEANI{A_i}$ e binary variables that are equal to 1 if the observation corresponds to the Americas, Asia or Southwest Pacific regions, respectively and 0 otherwise. $VENOUSBLOO{D_i}$ is equal to 1 if the blood sample was collected on venous blood (n=41) and 0 if collected on peripheral (i.e., finger prick) blood (n=215). Variation in the type of ICT used in surveys was captured through the covariate$\; TYPEIC{T_i}$, which is 1 for AMRAD ICT-based surveys (n=37) and 0 for surveys using the Binax test (n=218). We also fitted a model including random effects ${u_i}\sim \; N\; ({0,\; {\sigma^2}} )$ taking into account the variability not explained by the covariates. The deviance information criterion (DIC) was used to compare fitted models and eventually to determine model selection (i.e., model that yields the lowest DIC value was chosen).

## Results and Discussion

The literature search identified 264 surveys, including 86 surveys conducted prior to the implementation of MDA and 178 post-MDA surveys. Details of included studies are shown in Supplementary Table 1. In the majority (215/264) of studies mf was determined by thick smear using between 20 and 60 µl of blood; 36 studies used filtration techniques (using 1 to 3 ml blood). Four studies used both thick smear and filtration methods, with estimates obtained by filtration considered. None of the included surveys were conducted in areas where loiasis occurs or in areas where mass treatment against onchocerciasis had been implemented.

**Figure 1.**
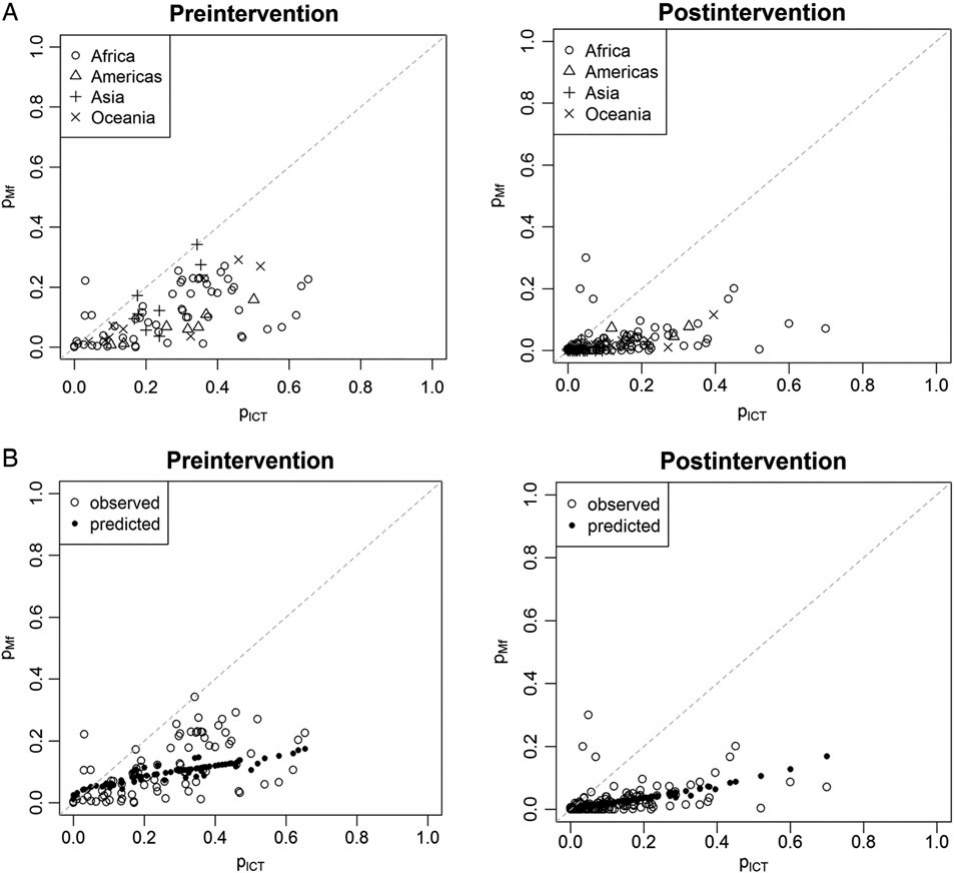
(A) The relationship between the prevalence of lymphatic filariasis based on microfilaraemia (p_mf_) and prevalence estimated from antigenemia (p_ICT_), grouped by continent and pre- and post-control. (B) Observed and predicted relationship between p_ICT_ and p_mf._ For each p_ICT_ value, observed and predicted p_mf_ values obtained from the model without random effects grouped by pre- and post-control. Details of included studies are shown in Supplementary Table 1.

Figure 1A presents the relationship between p_mf_ and p_ICT_, stratified by pre- and post-intervention settings and by continent, and shows that p_ICT_ consistently overestimates prevalence compared to p_mf_, especially in post-control settings. The correlation between p_mf_ and p_ICT_ is positive, but not very strong (Spearman's correlation coefficient of 0.62 and 0.59 under pre- and post-intervention settings, respectively). Despite these correlations, we were unable to obtain a well-fitting regression model between p_ICT_ and p_mf_ that would enable prediction of p_mf_ from p_ICT_ (Figure 1B). DIC values for the fitted models were 1163 and 2960 with and without random effects, respectively, indicating that effects of unmeasured characteristics that affect all individuals surveyed contribute to the observed variability. We were unable to account for age group, as surveys typically targeted all age groups (n=229/264). Sensitivity analysis explored separate models for different volumes of blood examined (20 µl or 20–60 µl), but showed that overall model fit did not improve (see results in the Supplementary information).

Our analysis highlights a disparity in prevalence estimates of microfilaraemia, based on blood smears, and of antigenaemia based on use of an ICT test. This result was to be expected as filarial antigen tests detect circulating antigen of worm adults and in pre-intervention settings, antigenaemia in children can develop up to 2-3 years before the appearance of microfilaraemia.^6^ Among treated populations, adult worms retain the capacity to resume production of microfilariae and rates of antigenaemia are known to decline more slowly following treatment.^4^ Other factors related to MDA delivery, including treatment coverage, time interval between rounds and number of rounds, may also influence differently observed levels of microfilaraemia and antigenaemia. In addition, the timing of night blood collection may introduce variation in the estimates of mf prevalence since the density of circulating mf fluctuates overnight,^7^ but unfortunately few studies report this information. We recognize that our analysis lacked a gold standard of true LF infection and that technical and human factors may have influenced the reliability of both microscopy and ICTs,^2^ but the results clearly show that estimates of microfilaraemia and antigenaemia do not correspond in a predictable manner, especially in post-intervention settings. What was particularly surprising in our analysis was the large difference, up to 40–70%, in estimates of microfilaraemia and antigenaemia. This finding has a number of practical and scientific implications. First, WHO recommends that MDA is initiated in settings where the prevalence of LF >1% and whether this is based on p_mf_ (as initially recommended) or p_ICT_ (as currently recommended) may result in different treatment decisions. Second, the lack of a predictable relationship between microfilaraemia and antigenaemia means that they cannot be combined in studies that seek to develop risk maps of LF and future work should give careful consideration to the diagnostic method when predicting the spatial distribution of LF transmission.

## Supplementary data

Supplementary data are available at Transactions Online (http://trstmh.oxfordjournals.org/).

Supplementary Data
