# Supplementary material for: An investigation of the disparity in estimates of microfilaraemia and antigenaemia in lymphatic filariasis surveys
Source: Trans R Soc Trop Med Hyg. 2015 Jun 21;109(8):529–31. doi: 10.1093/trstmh/trv048 (PMC4542699; doi:10.1093/trstmh/trv048)
Supplement: Supplementary Data [file supp_109_8_529__index.html]

An investigation of the disparity in estimates of microfilaraemia and antigenaemia in lymphatic filariasis surveys — An investigation of the disparity in estimates of microfilaraemia and antigenaemia in lymphatic filariasis surveys — Supplementary Data 

# An investigation of the disparity in estimates of microfilaraemia and antigenaemia in lymphatic filariasis surveys

## Supplementary Data

Supplementary Data

- Supplementary Data - Docx file
