# Supplementary material for: An investigation of the disparity in estimates of microfilaraemia and antigenaemia in lymphatic filariasis surveys
Source: Trans R Soc Trop Med Hyg. 2015 Jun 21;109(8):529–31. doi: 10.1093/trstmh/trv048 (PMC4542699; doi:10.1093/trstmh/trv048)
Supplement: Supplementary Data [file supp_trv048_trv048supp.docx]

**Table 1.** Characteristics of lymphatic filariasis prevalence studies where microfilaraemia (pMF) and antigenemia (pICT) were assessed in the same sample population

| **Continent** | **Study** | **Country** | **Pre-control/**  **post-control** | **Population**  **surveyed mf** | **pMF** | **Population**  **surveyed ICT** | **pICT** |
| --- | --- | --- | --- | --- | --- | --- | --- |
|  |  |  |  |  | **median (IQR)** |  | **median (IQR)** |
| Africa | ^a^Benin MoH, 2002^1^ | Benin | 0/1 | 519 | 0.4 | 519 | 0 |
|  | ^a^Burkina Faso MoH, 2010^2^ | Burkina Faso | 6/3 | 3478 | 7.4 (2.3-8.7) | 2520 | 18.8 (10.1-20.7) |
|  | ^a^Cote d’Ivoire, 2009^3^ | Cote d'Ivoire | 3/0 | 794 | 1.9 (1.2-2.5) | 230 | 16.9 (1.2-17.3) |
|  | Ramzy et al., 1999^4^ | Egypt | 0/4 | 1813 | 2.9 (1.9-6.6) | 1813 | 8.2 (4.4-12.7) |
|  | Ramzy et al., 2006^5^ | Egypt | 10/2 | 9127 | 1.3 (0.3-2.9) | 11134 | 5 (3-12.1) |
|  | Geshere et al., 2012^6^ | Ethiopia | 0/1 | 123 | 0 | 60 | 0 |
|  | ^a^Ghana MoH, 2001^7^ | Ghana | 0/9 | 915 | 20 (18-23) | 915 | 40 (37-42) |
|  | Simonsen et al., 2002^8^ | Kenya | 0/1 | 1013 | 2.7 | 1013 | 16.5 |
|  | Njenga et al., 2011^9^ | Kenya | 8/8 | 2498 | 10.6 (1-22.2) | 2505 | 21.6 (10.5-33.9) |
|  | Nielsen et al., 2002^10^ | Malawi | 0/2 | 537 | 21.5 (20.3-22.6) | 507 | 64.4 (63.4-65.4) |
|  | ^a^Niger MoH, 2008^10^ | Niger | 0/2 | 984 | 0.9 (0.4-1.4) | 150 | 21.5 (17-26) |
|  | ^a^Niger MoH, 2009^11^ | Niger | 2/0 | 1075 | 0.3 (0.2-0.4) | 150 | 34.5 (17-52) |
|  | ^a^Nigeria MoH, 2004^12^ | Nigeria | 7/2 | 2726 | 1.8 (1.7-7.4) | 2726 | 15.3 (1.8-22.9) |
|  | ^a^Nigeria MoH, 2006^13^ | Nigeria | 9/0 | 1163 | 0.7 (0.5-2.5) | 1163 | 0.7 (0.5-2.5) |
|  | ^a^Nigeria MoH, 2008^14^ | Nigeria | 10/0 | 1013 | 0 (0-2.7) | 1013 | 7.4 (1.8-9.8) |
|  | ^a^Nigeria MoH, 2009^15^ | Nigeria | 3/0 | 1472 | 1.8 (1.2-3.1) | 0 | 0 |
|  | ^a^Nigeria MoH, 2010^16^ | Nigeria | 0/3 | 1294 | 10.8 (2.9-12.3) | 50 | 0 (0-46) |
|  | Richards et al., 2011^17^ | Nigeria | 34/10 | 9897 | 1.1 (0-3.9) | 9332 | 10.1 (5.5-22.3) |
|  | Ekanem et al., 2011^18^ | Nigeria | 0/1 | 222 | 0 | 222 | 17.1 |
|  | Ebenezer et al., 2011^19^ | Nigeria | 0/1 | 1803 | 7 | 1803 | 11.3 |
|  | ^a^Togo MoH, 2001^20^ | Togo | 0/7 | 3500 | 1.2 (0.8-10.6) | 615 | 4.9 (2.7-31.3) |
|  | ^a^Togo MoH, 2008^21^ | Togo | 3/0 | 1501 | 0 (0-2) | 1785 | 2.6 (0.3-8.4) |
|  | Meyrowitsch et al., 2004^22^ | Tanzania | 2/0 | 1024 | 18.4 (16.7-20.1) | 1024 | 44.3 (43.5-45.1) |
|  | Onapa et al., 2001b^23^ | Uganda | 0/1 | 173 | 0 | 289 | 0 |
|  | Onapa et al., 2001a^24^ | Uganda | 0/3 | 1257 | 22.4 (9.7-25.5) | 2686 | 29.1 (18.3-30.1) |
|  | ^a^Uganda MoH, 2010^25^ | Uganda | 4/0 | 2062 | 0.2 (0-0.5) | 454 | 0 |
|  | Ashton et al., 2011^26^ | Uganda | 14/0 | 938 | 4.7 (0-8.7) | 1398 | 13 (3.4-37.5) |
| Americas | Aguiar-Santos et al., 2013^27^ | Brazil | 0/1 | 159 | 1.2 | 159 | 13.8 |
|  | Braga et al., 2003^28^ | Brazil | 0/1 | 625 | 6.8 | 625 | 31.7 |
|  | Braga et al., 2005^29^ | Brazil | 0/1 | 1130 | 6.9 | 790 | 25.7 |
|  | De Rochars et al., 2005^30^ | Haiti | 0/4 | 455 | 9.5 (7.4-13.5) | 455 | 35.7 (22.4-43.5) |
|  | Boyd et al., 2010^31^ | Haiti | 6/0 | 3499 | 4.4 (4.2-7.4) | 3465 | 16.9 (11.8-28.7) |
| Asia | Ramaiah et al., 2007b^32^ | India | 0/1 | 238 | 17.2 | 57 | 17.5 |
|  | Chhotray et al., 2005^33^ | India | 0/2 | 4297 | 10.3 (9.5-11) | 2400 | 17.3 (16.8-17.8) |
|  | Sunish et al., 2001^34^ | India | 0/1 | 3505 | 12.2 | 3505 | 23.7 |
|  | Ramaiah & Vanamail, 2013^35^ | India | 3/0 | 415 | 0.4 (0-3.6) | 415 | 3.6 (0-4) |
|  | Chandrasena et al., 2002^36^ | Sri Lanka | 0/2 | 226 | 30.8 (27.5-34.2) | 226 | 34.8 (34.2-35.3) |
|  | Gunawardena et al., 2007^37^ | Sri Lanka | 24/0 | 4008 | 0 | 4008 | 1.4 (0-3.7) |
|  | Bhumiratana et al., 1999^38^ | Thailand | 0/1 | 225 | 5.8 | 225 | 20 |
|  | Bhumiratana et al., 2002^39^ | Thailand | 0/1 | 219 | 3.7 | 219 | 23.7 |
| Oceania | ^b^WHO Country report | Fiji | 4/0 | 6762 | 0.7 (0.2-1.6) | 6762 | 7 (1.9-13.3) |
|  | WHO Annual report, 2003^40^ | New Caledonia | 0/1 | 382 | 3.6 | 382 | 32.5 |
|  | Fraser et al., 2005^41^ | Vanuatu | 10/8 | 2079 | 2.2 (0-7.1) | 2079 | 10.2 (3.9-27.2) |
|  | Ichimori et al., 2007^42^ | Samoa | 1/0 | 4054 | 1.1 | 4054 | 4.2 |
|  | Joseph et al., 2011^43^ | Samoa | 7/0 | 1269 | 0.5 (0.2-0.6) | 1269 | 14.6 (8.4-16.7) |
|  | ^c^WHO Country report | Samoa | 7/0 | 6448 | 0.4 (0.2-0.8) | 6448 | 2.1 (0.7-3) |

ICT: immuno-chromatographic test; mf: microfilariae; IQR = inter-quartile range.

^a^ Unpublished data.

^b^ http://www.wpro.who.int/southpacific/pacelf/countries/fji/activities/en/index.html.

^c^ http://www.wpro.who.int/southpacific/pacelf/countries/wsm/activities/en/index.html.

References

1. Benin MoH. Programme to Eliminate Lymphatic Filariasis. Country Report WHO; BEN0004_LF2002.Cotonou: Benin Ministry of Health; 2002.

2. Burkina Faso MoH. Programme to Eliminate Lymphatic Filariasis. Country Report WHO; BFA0020_LF2010. Ouagadougou: Burkina Faso Ministry of Health; 2010.

3. Cote d’Ivoire MoH; Programme to Eliminate Lymphatic Filariasis. Country Report WHO; CIV0003_LF2009. Abidjan: Cote d'Ivoire Ministry of Health; 2009.

4. Ramzy RM, Helmy H, el-Lethy AS et al. Field evaluation of a rapid-format kit for the diagnosis of bancroftian filariasis in Egypt. East Mediterr Health J 1999;5:880-7.

5. Ramzy RM, El Setouhy M, Helmy H et al. Effect of yearly mass drug administration with diethylcarbamazine and albendazole on bancroftian filariasis in Egypt: a comprehensive assessment. Lancet 2006;367:992-9.

6. Geshere Oli G, Tekola Ayele F, Petros B. Parasitological, serological and clinical evidence for high prevalence of podoconiosis (non-filarial elephantiasis) in Midakegn district, central Ethiopia. Trop Med Int Health 2012;17:722-6.

7. Ghana MoH. Programme to Eliminate Lymphatic Filariasis. Country Report WHO;GHA0020_LF2001. Accra: Ghana Ministry of Health; 2001.

8. Simonsen PE, Meyrowitsch DW, Jaoko WG et al. Bancroftian filariasis infection, disease, and specific antibody response patterns in a high and a low endemicity community in East Africa. Am J Trop Med Hyg 2002;66:550-9.

9. Njenga SM, Mwandawiro CS, Wamae CN et al. Sustained reduction in prevalence of lymphatic filariasis infection in spite of missed rounds of mass drug administration in an area under mosquito nets for malaria control. Parasit Vectors 2011;4:90.

10. Nielsen NO, Makaula P, Nyakuipa D et al. Lymphatic filariasis in Lower Shire, southern Malawi. Trans R Soc Trop Med Hyg 2002;96:133-8.

11. Nigeria MoH. Programme to Eliminate Lymphatic Filariasis. Country Report WHO; NER0009_LF2009. Abuja: Niger Ministry of Health; 2009.

12. Nigeria MoH. Programme to Eliminate Lymphatic Filariasis. Country Report WHO; NGA0004_LF2004. Abuja: Nigeria Ministry of Health; 2004.

13. Nigeria MoH. Programme to Eliminate Lymphatic Filariasis. Country Report WHO; NGA0007_LF2006. Abuja: Nigeria Ministry of Health; 2006.

14. Nigeria MoH. Programme to Eliminate Lymphatic Filariasis. Country Report WHO; NGA0013_LF2008. Abuja: Nigeria Ministry of Health; 2008.

15. Nigeria MoH. Programme to Eliminate Lymphatic Filariasis. Country Report WHO; NGA0014_LF2009. Abuja: Nigeria Ministry of Health; 2009.

16. Nigeria MoH. Programme to Eliminate Lymphatic Filariasis. Country Report WHO; NGA0016_LF2010. Abuja: Nigeria Ministry of Health; 2010.

17. Richards FO, Eigege A, Miri ES et al. Epidemiological and entomological evaluations after six years or more of mass drug administration for lymphatic filariasis elimination in Nigeria. PLoS Negl Trop Dis 2011;5:e1346.

18. Ekanem IA, Alaribe AAA, Ekanem AP. Prevalence of Bancroftian Filariasis among Edim Otop sub-urban dwellers in Calabar Municipality of Cross River state, Nigeria. J Appl Pharm Sci 2011;01.

19. Ebenezer A, Amadi EC, Agi PI. Studies on the microfilaria, antigenemia and clinical signs of bancroftian filariasis in Epie creek communities, Niger Delta, Nigeria. Int Res J Microbiol 2011;2:370–4.

20. Togo MoH. Programme to Eliminate Lymphatic Filariasis. Country Report WHO; TGO0003_LF2001. Lome: Togo Ministry of Health; 2001.

21. Togo MoH. Programme to Eliminate Lymphatic Filariasis. Country Report WHO; TGO0015_LF2008. Lome: Togo Ministry of Health; 2008.

22. Meyrowitsch DW, Simonsen PE, Magesa SM. A 26-year follow-up of bancroftian filariasis in two communities in north-eastern Tanzania. Ann Trop Med Parasitol 2004;98:155-69.

23. Onapa AW, Simonsen PE, Pedersen EM. Non-filarial elephantiasis in the Mt. Elgon area (Kapchorwa District) of Uganda. Acta Trop 2001;78:171-6.

24. Onapa AW, Simonsen PE, Pedersen EM et al. Lymphatic filariasis in Uganda: baseline investigations in Lira, Soroti and Katakwi districts. Trans R Soc Trop Med Hyg 2001;95:161-7.

25. Uganda MoH. Programme to Eliminate Lymphatic Filariasis. Country Report WHO; UGA0014_LF2010. Kampala: Uganda Ministry of Health; 2010.

26. Ashton RA, Kyabayinze DJ, Opio T et al. The impact of mass drug administration and long-lasting insecticidal net distribution on *Wuchereria bancrofti* infection in humans and mosquitoes: an observational study in northern Uganda. Parasit Vectors 2011;4:134.

27. Aguiar-Santos AM, Medeiros Z, Bonfim C et al. Epidemiological assessment of neglected diseases in children: lymphatic filariasis and soil-transmitted helminthiasis. J Pediatr (Rio J) 2013;89:250-5.

28. Braga C, Dourado MI, Ximenes RA et al. Field evaluation of the whole blood immunochromatographic test for rapid bancroftian filariasis diagnosis in the northeast of Brazil. Rev Inst Med Trop Sao Paulo 2003;45:125-9.

29. Braga C, Dourado I, Ximenes R et al. Bancroftian filariasis in an endemic area of Brazil: differences between genders during puberty. Rev Soc Bras Med Trop 2005;38:224-8.

30. de Rochars MB, Kanjilal S, Direny AN et al. The Leogane, Haiti demonstration project: decreased microfilaremia and program costs after three years of mass drug administration. Am J Trop Med Hyg 2005;73:888-94.

31. Boyd A, Won KY, McClintock SK et al. A community-based study of factors associated with continuing transmission of lymphatic filariasis in Leogane, Haiti. PLoS Negl Trop Dis 2010;4:e640.

32. Ramaiah KD, Vanamail P, Das PK. Changes in Wuchereria bancrofti infection in a highly endemic community following 10 rounds of mass administration of diethylcarbamazine. Trans R Soc Trop Med Hyg 2007;101:250-5.

33. Chhotray GP, Ranjit MR, Khuntia HK et al. Precontrol observations on lymphatic filariasis & geo-helminthiases in two coastal districts of rural Orrisa. Indian J Med Res 2005;122:388-94.

34. Sunish IP, Rajendran R, Satyanarayana K et al. Immunochromatographic test (ICT) for estimation of true prevalence of bancroftian filariasis in an endemic area in southern India. Trans R Soc Trop Med Hyg 2001;95:607-9.

35. Ramaiah KD, Vanamail P. Surveillance of lymphatic filariasis after stopping ten years of mass drug administration in rural communities in south India. Trans R Soc Trop Med Hyg;107:293-300.

36. Chandrasena TGAN, Premaratnal R. Evaluation of the ICT whole-blood *Wuchereria bancrofii* in Sri Lanka card test to detect infection due to. Trans R Soc Trop Med Hyg 2002;96:60-3.

37. Gunawardena GSa, Ismail MM, Bradley MH et al. Impact of the 2004 mass drug administration for the control of lymphatic filariasis, in urban and rural areas of the Western province of Sri Lanka. Ann Trop Med Parasitol 2007;101:335-41.

38. Bhumiratana A, Koyadun S, Suvannadabba S et al. Field trial of the ICT filariasis for diagnosis of *Wuchereria bancrofti* infections in an endemic population of Thailand. Southeast Asian J Trop Med Public Health 1999;30:562-8.

39. Bhumiratana A, Wattanakull B, Koyadun S et al. Relationship between male hydrocele and infection prevalences in clustered communities with uncertain transmission of *Wuchereria bancrofti* on the Thailand-Myanmar border. Southeast Asian J Trop Med Public Health 2002;33:7-17.

40. WHO. Global Programme to Eliminate Lymphatic Filariasis. Annual Report on Lymphatic Filariasis 2003. Geneva: World Health Organization; 2005.

41. Fraser M, Taleo G, Taleo F et al. Evaluation of the program to eliminate lymphatic filariasis in Vanuatu following two years of mass drug administration implementation: results and methodologic approach. Am J Trop Med Hyg 2005;73:753-8.

42. Ichimori K, Tupuimalagi-Toelupe P, Toeaso Iosia V et al. *Wuchereria bancrofti* filariasis control in Samoa before PacELF (Pacific Programme to Eliminate Lymphatic Filariasis). Trop Med Health 2007;35:261-9.

43. Joseph H, Maiava F, Naseri T et al. Epidemiological assessment of continuing transmission of lymphatic filariasis in Samoa. Ann Trop Med Parasitol 2011;105:567-78.

**Sensitivity analysis upon regression model**

We conducted a sensitivity analysis by fitting various models based solely on thick smears based on 1. 20 µl of blood volume; 2. 20 to 60 µl of blood (equivalent to 1-3 thick smears); and 3. 50 µl or 60 µl of blood, as follows:

for each survey $i=1,\ldots,N,$

$${Y_{{mf}_{i}}|p}_{{mf}_{i}}\sim Binomial (N_{{mf}_{i}},p_{{mf}_{i}})$$

**Model 1. Only thick smears based on 20 µl of blood**

$$\mathrm{logit}\left( p_{{Mf}_{i}} \right)= \alpha+\alpha_{1}\mathrm{logit}\left( p_{{ICT}_{i}} \right)+\alpha_{\mathrm{PRE}}\times{PRE}_{i}+\alpha_{1PRE}\mathrm{logit}\left( p_{{ICT}_{i}} \right)\times{PRE}_{i}$$


DIC = 863.9; DIC with random effects = 316.7

**Model 2. Thick smears based on 20-60 µl of blood**

$$\mathrm{logit}\left( p_{{Mf}_{i}} \right)= \alpha+\alpha_{1}\mathrm{logit}\left( p_{{ICT}_{i}} \right)+\alpha_{\mathrm{PRE}}\times{PRE}_{i}+\alpha_{1PRE}\mathrm{logit}\left( p_{{ICT}_{i}} \right)\times{PRE}_{i}$$


DIC = 2147.6; DIC with random effects = 858.3

**Model 3. Thick smears based on 20-60 µl of blood, and adjusting by blood volume**

$\mathrm{logit}\left( p_{{Mf}_{i}} \right)= \alpha+\alpha_{1}\mathrm{logit}\left( p_{{ICT}_{i}} \right)+\alpha_{\mathrm{PRE}}\times{PRE}_{i}+\alpha_{1PRE}\mathrm{logit}\left( p_{{ICT}_{i}} \right)\times{PRE}_{i}$ + $\alpha_{BLOOD50}\times\mathrm{BLOOD}_{50}+\alpha_{BLOOD60}\times\mathrm{BLOOD}_{60}$

DIC = 2041.8; DIC with random effects = 852.5

None of the new models improve the overall model fit and therefore we have not altered the results.
